# Book Reviews

**Published:** 1900-01

**Authors:** 


RECOLLECTIONS OF A REBEL SURGEON (AND OTHER SKETCHES); OR, IN THE DOCTOR'S SAPPY DAYS. By F. E. Daniel, M.D. Illustrated. 1899. Von Boeckmann, Schutze & Co., Austin, Texas.

In his Recollections of a Rebel Surgeon, Dr. F. E. Daniel, editor of the *Texas Medical Journal*, has published, in a book of 264

pages, a most charming and delightful collection of anecdotes and reminiscenses of war time. There is very little of the touch of the amateur about them, and one is never offended by rasping dialectic inaccuracies which are so prone to occur in sketches of this character. Besides stories of war time and camp life, there are others dealing with occurrences in the life of a country doctor. There is nothing dull in the book, and in reading it one feels as if he were being entertained by some lively and amusing companion. The anecdotes are generally humorous—good, wholesome, clean humor; some are pathetic, touching and tender. The book sells for the modest price of $1.10, postpaid, and can be obtained from the author at Austin, Texas. The purchaser will have a rare treat. James Whitcomb Riley or Opie Reed has produced nothing more readable than has Dr. Daniel in his Recollections, though the manner of both is suggested, not in imitation but in kinship of talent.

We are in receipt of the Physician's Visiting List for 1900, from the well-known house of P. Blakiston's Son & Co. It is neatly gotten up, as usual, and its arrangement exceedingly handy. This volume is always a welcome visitor.

---

### *MAGAZINE NOTES.*

---

*The Ladies' Home Journal* for 1899 has afforded its readers many hours of excellent reading on the most varied topics of the day. Its illustrations have been superb throughout. For 1900 we are informed that the work of its pages will surpass all previous years and prove more entertaining than ever. Every home should be a subscriber to this monthly periodical.

